# Clinical Cases

**Published:** 1887-08

**Authors:** G. W. Sherbino

**Affiliations:** Abilene, Texas


### G. W. Sherbino, M. D., Abilene, Texas.

*Neuralgia.*—Old lady who has had neuralgia for several years, and my success with her case was anything but flattering. I have not given her any medicine for a year. The symptoms were pain now in the left side, and then in the left shoulder; then leaves and goes to her head; from thence down her spine, and she was never easy. Came into office. (While she was relating to me her symptoms, *she was blowing her breath on her hands all the time.*)

I asked her what she was doing that for, and she said, "Because they burn so, and my feet are as hot as fire at night;" Lac. can., one dose, S. L. to follow. Came back in a few days saying that the last medicine had done her more good than all I had ever given her. When the symptoms came back another dose was given with the same happy results, and I think it will make a permanent cure in this almost hopeless case.

*Ovarian Neuralgia.*—Case I.—Miss —— has had hæmorrhoids for past year, and pain in the right ovarian region. The pain changes from the piles to this sore place in the side, then from the ovary back to the hæmorrhoids again. Restless at night, slept but very little. Burning in the right ovary. (Hands and feet abnormally hot and burn so at night.) "*Doctor, I want to lie with my knees to my chin.* I cannot get any relief in any other position." Lac. can. cured her and another friend who had the same peculiar position with the knees drawn up to the chin.

Case II.—Mrs. ——, visiting friends, was taken in the afternoon with bearing down in the uterus and left ovary. *Could not stand on her feet without holding up the abdomen.* Very distressing pain shooting at times down into the thigh. Pain came in paroxysms. She took to bed, lying on left side, with left limb somewhat flexed.

Two months after confinement she was taken with this same neuralgia, for which she received a hypodermic of Morphine. On the dorsal surface of the wrist an abscess formed, which did not heal for two months, leaving a scar that will last a lifetime.

"She got one dose dry," after which there was an aggravation. They came to tell me I would have to give Morphine. I sent more "S. L.," and she soon was relieved.

CASE III.—Mrs. ——, aged twenty-seven, mother of six children, has had poor health since her last confinement two years ago.

She is subject to spells of pain in the left ovary, and always got a "hypnotic" from a regular.

I was sent for late in the afternoon, but was away from home. I saw the case at nine P. M. She had bearing, forcing down pain, like the early stage of labor. *Pain starts in the left ovarian region. Shoots* down into the upper part of thigh. At the same time the pain shoots up the left side near the chest, strikes across the stomach,* and makes her sick. Aggravation from motion (Bry. alb., Baptis.) and many others. *Pain came quick, and when leaving went quick.* When pain came she would throw her head back and bend her body backward (Diosc.). Sensitive to jar. (Lili. tig., Lac. can.)

She was relieved by pressing down upon the abdomen with her hands during a pain.

Gave one dose, followed by S. L., every ten minutes. In half an hour "*no better, no worse.*" I believe it was right. Gave one more dose. In half an hour more pain left as sudden as it came, to return no more. Next morning she was able to do her housework. But after a hypnotic she generally kept her bed for a week.

